# Comparative Study of Multimodal Biometric Recognition by Fusion of Iris and Fingerprint

**DOI:** 10.1155/2014/829369

**Published:** 2014-01-29

**Authors:** Houda Benaliouche, Mohamed Touahria

**Affiliations:** Computer Science Department, University of Ferhat Abbas Sétif 1, Pôle 2 - El Bez, 19000 Sétif, Algeria

## Abstract

This research investigates the comparative performance from three different approaches for multimodal recognition of combined iris and fingerprints: classical sum rule, weighted sum rule, and fuzzy logic method. The scores from the different biometric traits of iris and fingerprint are fused at the matching score and the decision levels. The scores combination approach is used after normalization of both scores using the min-max rule. Our experimental results suggest that the fuzzy logic method for the matching scores combinations at the decision level is the best followed by the classical weighted sum rule and the classical sum rule in order. The performance evaluation of each method is reported in terms of matching time, error rates, and accuracy after doing exhaustive tests on the public CASIA-Iris databases V1 and V2 and the FVC 2004 fingerprint database. Experimental results prior to fusion and after fusion are presented followed by their comparison with related works in the current literature. The fusion by fuzzy logic decision mimics the human reasoning in a soft and simple way and gives enhanced results.

## 1. Introduction

Biometrics refers to identity verification of persons according to their physical or behavioral characteristics. Many physical body parts and personal features have been used for biometric systems: fingers, hands, feet, faces, irises, retinas, ears, teeth, veins, voices, signatures, typing styles, gaits, odors, and DNA. Person verification based on biometric features has attracted more attention in designing security systems [1]. However, no single biometrical feature can meet all the performance requirements in practical systems [2]. Most of biometric systems are far from satisfactory in terms of user confidence and user friendliness and have a high false rejection rate FRR. There is a need for development of novel paradigms and protocols and improved algorithms for human recognition. Unimodal biometric systems use one biometric trait to recognize individuals. These systems are far from perfect and suffer from several problems like noise, nonuniversality, lack of individuality, and sensitivity to attack. Multimodal biometric systems use multiple modalities to overcome the limitations that arise when using single biometric trait to recognize individuals. Multiple biometric systems perform better than unimodal biometric systems. The use of only one biometric trait susceptible to noise, bad capture, and other inherent problems makes the unimodal biometric system unsuited for all applications.

Many works in the literature have demonstrated that the drawbacks of the unimodal biometric systems are mainly genuine and imposters identification failure due to the intraclass variations and the interclass similarities, while the drawbacks associated with multimodal biometrics are increased complicity of the system with two or more sensors [2–6] and thus higher cost, as well as inconvenience of using several biometrics. So, identification of person with high accuracy and less complexity of the system is becoming critical in a number of security issues in our society. Iris and fingerprint biometrics are more simple, accurate, and reliable as compared to other available traits. These properties make their fusion particularly promising solution to the authentication problems today. Moreover, fusion of iris and fingerprint is more reliable than fusion of each one with another biometric like face [7]. However, iris biometric has more features and stability and resistance to attacks than fingerprint biometric; despite this, the conventional fusion methods still use the same weight in fusion for each single biometric, and this is the reason for why their best error rates are far from perfect. False accept rate identifies the number of times an imposter is classified as a genuine user by the system and false reject rate pertains to misidentification of a genuine user as an imposter. Although ideally both FAR and FRR should be as close to zero as possible in real systems, however, this is not the case [8]. For an ideal authentication system, FAR and FRR indexes are equal to 0. To increase the related security level, system parameters are then fixed in order to achieve the FAR = 0% point and a corresponding FRR point [9].

In this paper a novel combination of iris and fingerprint biometrics is presented in order to achieve best compromise between a zero FAR and its corresponding FRR; in our approach, iris trait has more weight in fusion with fingerprint and the system decision is made to have more intermediate values between bad and good recognition; the weight is simply an appreciation we assign to the matching distance for each single biometric set by fuzzy membership function and we use major concepts of fuzzy logic introduced by Zadeh [10] which are fuzzy sets, fuzzy membership function, and fuzzy inference system. The fuzzy inference system mimics our human thinking and this is mainly the reason we get enhanced results.

The objective of this research is threefold: first designing and implementing monomodal systems for the biometric recognition of iris and fingerprint, these systems will serve latter for comparison; second, designing and implementing a multimodal biometric system of combined iris and fingerprint using the previous monomodal systems with three different matching algorithms, two classical matching algorithms and our proposed one based on fuzzy logic; third carrying out exhaustive and intensive tests on the iris and fingerprint databases using the proposed recognition schemes to conclude at the end the best one. At last, a comparison of the achieved results with similar works in the current literature is given.

The paper is organized as follows: in the next section related works are presented followed by a presentation of state of the art of multimodal biometric; in Section 3 the work methodology is presented and the two modalities are combined through three different experiments using two levels of fusion, one at the score level and the other at the decision level; in Section 4 the details of the system implementation is given and the databases involved in the work are presented; the experimental results prior to fusion and after fusion are presented in Section 5 followed by their evaluation and comparison in Section 6. Conclusion is given in the last section.

## 2. Related Works

Multimodal biometrics has been proposed by Ross and Jain in 2003 [11]. The concept of biometric multimodalities fusion is introduced with different fusion strategies and various levels of fusion are also presented [2, 4, 6, 12–17]. Fusion of iris and fingerprint has attracted a lot of attention and researchers have presented variety of approaches in the literature [7, 8, 16, 18, 19]. Baig et al. [8] in 2009 proposed a framework for multimodal biometric fusion based on utilization of a single matcher implementation for both modalities (iris and fingerprint). For their experiment they used the West Virginia University's multimodal database containing 400 images (4 enrolment images × 100 users) and the threshold is set to the equal error rate EER. The comparison is being made in terms of percentage improvement in EER rather than the EER values themselves. Jagadeesan et al. [16] in 2010 introduced a technique for cryptographic key generation by fusing fingerprint and iris biometrics. The fingerprint extractor is minutia based while the iris extractor is based on canny edge detector and Hough transform (Daugman's approach). The minutiae points and texture properties were first extracted from fingerprint and iris images, respectively, and then they were fused at the feature level to obtain the multibiometric template and subsequently a 256-bit secure cryptographic key from the multibiometric template is generated.

In 2011, Jameer Basha et al. [18] introduced a new framework for iris and fingerprint fusion at rank level; they conducted experimental tests using three implemented fusion methods: highest rank method, Borda count method, and logistic regression method. Their work achieved the best execution time required to match which is equal to 0.45 seconds for the highest rank method with optimal FAR and FRR equal, respectively to 0% and 0.25%.

In 2012, Radha and Kavitha [19] presented a novel fusion scheme of fingerprint and iris modalities at feature extraction level. The scheme uses a concatenated feature vector from both iris and fingerprint. The log Gabor filter is used to extract the feature vectors of both modalities. then the phase data from 1D log Gabor filters is extracted and quantized to four levels to encode the unique pattern of iris and Fingerprint into bitwise biometric template. Hamming distance (HD) is used to generate a final match score. Experimental results were verified on database of 50 users accounting to FAR = 0% and FRR = 4.3%. The execution time required to match is reduced to 0.14 seconds.

In 2013, Abdolahi et al. [7] presented a multimodal biometric system (fingerprint and iris) using fuzzy logic and weighted code. After converting fingerprint and iris images to a binary code, the decision level fusion is used to combine the results. Fingerprint code is weighed as 20% and iris code as 80%. The work achieved 2% FAR and FRR and 98.3% accuracy.

## 3. State of the Art of Multimodal Biometric

In this section we summarize the main ideas and principles involved in the area of multimodal biometric recognition.

### 3.1. Multimodal Biometrics versus Multibiometrics

As explained by most research papers in the field of biometric recognition [5, 12, 16, 20], the term “multimodal biometric” refers to multiple biometric traits used together at a specific level of fusion to recognize persons. The “multibiometrics” includes either the use of multiple algorithms, also called classifiers at enrolment or matching stages for the same biometric trait, or the use of multiple sensors of the same biometric trait like using different instruments to capture the biometric details, or using multiple instances of the same biometric trait like the use of fingerprints of three fingers, or finally using repeated instances like repeated impressions of one finger.

### 3.2. Fusion in Biometry

**Figure 1:**
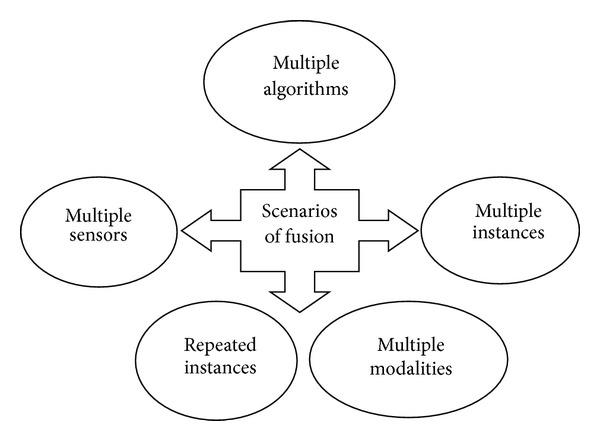
Fusion scenarios of multimodal systems.

In order to join two or more biometric traits, a method called “fusion” is used [12]. Fusion in biometry refers to the process of combining two or more biometric modalities. In this section we present the different scenarios of fusion used by multimodal biometric systems. It is worth noting that the multimodality does not involve the use of multiple biometric modalities in the strict sense of the term (such as combining iris and fingerprint), but its meaning is broader as defined in the following by the various scenarios of fusion (see Figure 1).

#### 3.2.1. Level of Fusion

**Figure 2:**
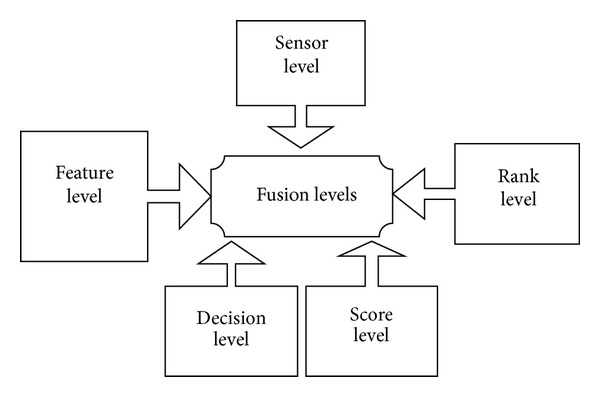
Levels of fusion in multimodal biometric systems.

Five levels of fusion in multimodal systems were introduced in the literature [4, 12] which are the following.

(*1) Sensor Level*. Multisensorial biometric systems sample the same instance of a biometric trait with two or more distinctly different sensors [14]. Processing of the multiple samples can be done with one algorithm or combination of algorithms. Example face recognition application could use both a visible light camera and an infrared camera coupled with specific frequency.

(*2) Feature Level*. The feature level fusion is useful in classification [14]. Different feature vectors are combined, obtained either with different sensors or by applying different feature extraction algorithms to the same raw data [21].

(*3) Decision Level*. With this approach, each biometric subsystem completes autonomously the processes of feature extraction, matching, and recognition. Decision strategies are usually of Boolean functions, where the recognition yields the majority decision among all present subsystems [9].

(*4) Rank Level*. Instead of using the entire template, partitions of the template are used. Ranks from template partitions are consolidated to estimate the fusion rank for the classification [18]. Rank level fusion involves combining identification ranks obtained from multiple unimodal biometrics. It consolidates a rank that is used for making final decision [19].

(*5) Score Level*. It refers to the combination of matching scores provided by the different systems. The score level fusion techniques are divided into two main sets: fixed rules (AND, OR, majority, maximum, minimum, sum, product and arithmetic rules) and trained rules (weighted sum, weighted product, fisher linear discriminate, quadratic discriminate, logistic regression, support vector machine, multilayer perceptrons, and Bayesian classifier ) [22].  Figure 2 shows the five levels of biometric fusion.

#### 3.2.2. Normalization

Score normalization brings both matching scores between 0 and 1 [23]. The normalization of both scores by the min-max rule are given by

(1) $$\begin{array}{c} N_{\text{Iris}}=\frac{\text{M}{\text{S}}_{\text{Iris}}-\text{mi}{\text{n}}_{\text{Iris}}}{\text{ma}{\text{x}}_{\text{Iris}}-\text{mi}{\text{n}}_{\text{Iris}}}, \end{array}$$

(2) $$\begin{array}{c} N_{\text{Finger}}=\frac{\text{M}{\text{S}}_{\text{Finger}}-\text{mi}{\text{n}}_{\text{Finger}}}{\text{ma}{\text{x}}_{\text{Finger}}-\text{mi}{\text{n}}_{\text{Finger}}}, \end{array}$$

where MS_Iris_ and MS_Finger_ are the matching scores obtained from iris and fingerprint modalities, respectively. min⁡_Iris_ and max⁡_Iris_ are the minimum and maximum scores for iris recognition and min⁡_Finger_ and max⁡_Finger_ are the corresponding values obtained from fingerprint trait. Other normalization algorithms also exist, like *Z*-score, TanH and Sigmoid which gave very good results. TanH method gave the best result but it involved a lot of parameters. *Z*-score and min-max are simple but they are insensitive to the presence of outliers [17].

## 4. The Research Methodology

**Figure 3:**
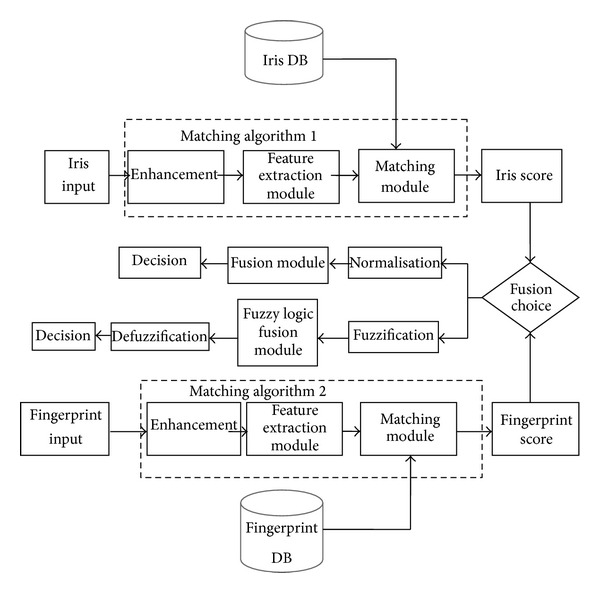
Flow chart of the application showing the main modules of the multimodal biometric recognition system.

Figure 3 shows the different stages included in our multimodal recognition system and the overall system design shows the following.

- The level at which the biometric information of the iris and fingerprint are fused is indicated (here two levels are used: the score level fusion is used for the classical fusion and the decision level fusion is used for the fusion with fuzzy logic).
- The fusion approach used is the approach by combining scores when the method of fusion is classic.
- The other fusion approach used is fusion of decisions when the method of fusion is fuzzy.
- The normalization of scores is required prior to the fusion only for the classical fusion (which is explained by the use of the approach by combining scores for both classical sum rule matching and matching by the linear weighted sum rule).
- Fusion by fuzzy logic does not require normalization of scores and only decisions are used by the fuzzy inference system.
- Three matching algorithms are used: the classical sum rule matching, the weighted sum rule matching, and our proposed matching with fuzzy logic.

The conventional fusion methods [2–4, 9, 11–17] use the same weight for each single biometric trait, but some biometric traits are more precise than the other ones; they have more stability and resistance to attacks. So in our approach, iris trait has more weight in fusion with fingerprint. Weight here is not a number assigned to the matching score, but a decision with intermediate values related to the matching distance.

**Figure 4:**
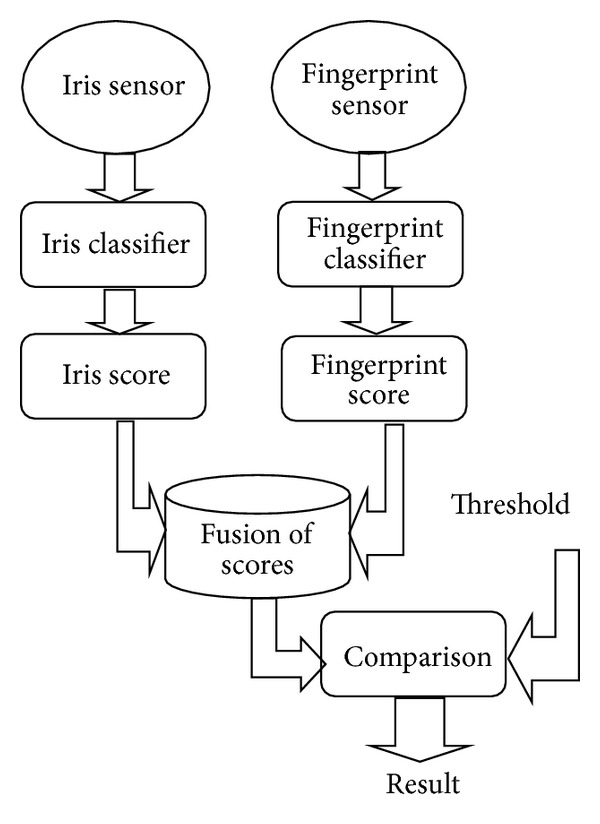
Score level fusion using iris and fingerprint biometric modalities.

**Figure 5:**
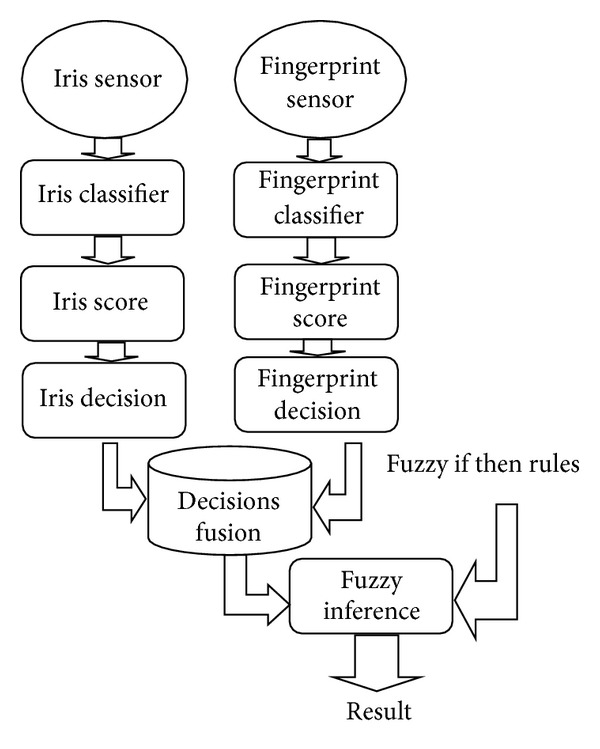
Decision level fusion using iris and fingerprint biometric modalities.

In this work, we have implemented two different architectures of the combined iris and fingerprint biometric recognition system in order to compare the recognition results (in terms of time, accuracy, and error rates) of both system architectures and conclude the best one. The first system architecture (see Figure 4) is based on the classical fusion of scores. The second system architecture (see Figure 5) is based on our proposed fuzzy logic matching using iris and fingerprint decisions.

### 4.1. Classical Matching Strategies

#### 4.1.1. Hamming Distance Based Matching

For the iris modality we use the hamming distance based matching:

(3) $$\begin{array}{c} \text{HD}=\frac{\sum_{j=1}^{N}X_{j}\left(\text{XOR}\right)Y_{j}\left(\text{AND}\right)Xn_{j}^{\prime }\left(\text{AND}\right)Yn_{j}^{\prime }}{N-\sum_{k=1}^{N}Xn_{k}\left(\text{OR}\right)Yn_{k}}. \end{array}$$

The hamming distance HD is calculated using formula (3), where *X*_*j*_ and *Y*_*j*_ are the models to compare bit by bit, *Xn*_*j*_ and *Yn*_*j*_ are the noise masks for *X*_*j*_ and *Y*_*j*_, and *N* is the number of bits represented by each model. For the fingerprint modality we use the Euclidian distance based matching (see formula (4)):

(4) $$\begin{array}{c} \text{ED}=\sqrt{\sum_{i=1}^{n}{\left(X_{i}-Y_{i}\right)}^{²}}, \end{array}$$

where *X*_*i*_ and *Y*_*i*_ are the models to compare.

#### 4.1.2. The Sum Rule Based Matching

After the normalization of both iris and fingerprint scores, the score of fusion is calculated as presented by formula (5):

(5) $$\begin{array}{c} S^{\prime }=\sum_{k=1}^{n}S, \end{array}$$

where *S*′ is the score of fusion and *n* is the number of the scores, here *n* = 2.

#### 4.1.3. The Weighted Sum Rule Based Matching

After the normalization of fingerprint and iris scores, the score of fusion is calculated as presented by formula (6):

(6) $$\begin{array}{c} S^{\prime }=\alpha S_{1}+\left(1-\alpha \right)S_{2}, \end{array}$$

where *S*′ is the score of fusion,  *S*_1_  and  *S*_2_  are, respectively, the scores of the biometric modalities to be combined, and *α* is the weight assigned to each modality.

In our experimentation we set *α* to 0.8 for the iris modality and 1 − *α* = 0.2 for the fingerprint modality.

### 4.2. Fuzzy Logic Based Matching

Our proposed fuzzy matching algorithm assigns a specific appreciation to each decision according to the best threshold minimizing both FRR and FAR. The fuzzy if-then rules produce decisions according to the matching distance calculated for each modality.

For that,

- we define two fuzzy variables for the input: “finger” for the fingerprint trait and “iris” for the iris trait,
- we define the output fuzzy variable: “fusion,”
- each variable is represented by a trapezoidal fuzzy set,
- for the inputs, we define three fuzzy sets according to the matching distance: bad, medium, and good,
- the output is fuzzy: either very bad or bad or medium or good or very good, or excellent.

**Figure 6:**
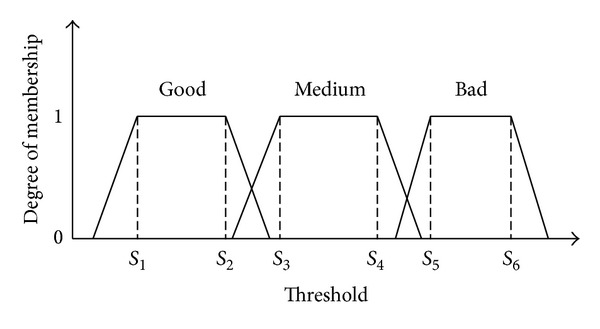
Fuzzy sets of the proposed entries and their trapezoidal membership functions.

As shown by Figure 6, [*S*_1_, *S*_2_] is the interval of thresholds belonging to the fuzzy set “good.” [*S*_3_, *S*_4_] is the interval of thresholds belonging to the fuzzy set “medium.” [*S*_5_, *S*_6_] is the interval of thresholds belonging to the fuzzy set “bad.”

The fuzzy if-then rules: combining decisions from iris and fingerprint modalities respect the following fuzzy rules:

If (finger is bad) and (iris is bad) then (fusion is very bad)
If (finger is bad) and (iris is medium) then (fusion is medium)
If (finger is bad) and (iris is good) then (fusion is good)
If (finger is medium) and (iris is bad) then (fusion is bad)
If (finger is medium) and (iris is medium) then (fusion is good)
If (finger is medium) and (iris is good) then (fusion is very good)
If (finger is good) and (iris is bad) then (fusion is medium)
If (finger is good) and (iris is medium) then (fusion is very good)
If (finger is good) and (iris is good) then (fusion is excellent).

We have set the if-then rules according to the following criteria:

- the iris decision is more reliable than the fingerprint decision, so we give more weight to the iris decision in fusion with the fingerprint decision,
- the fusion decision is one of the following sets: very bad, bad, medium, good, very good, excellent,
- in the cases where the iris decision is “bad,” the fusion decision should be either “bad” or “very bad” or “medium” even if the fingerprint decision is good,
- in the cases where the iris decision is “good,” the fusion decision should be either “good” or “excellent” even if the fingerprint decision is “bad.”

## 5. System Implementation

The programming language used to implement our system is MATLAB 7.10.0(R2010a). MATLAB as well as its interactive environment is a high-level language that allows the execution of tasks requiring high computing power and whose implementation will be much easier and faster than with traditional programming languages such as C, C++. It has several toolkits in particular image processing “Image Processing Toolbox” which proposes a set of algorithms and graphical reference tools for the processing, analysis, visualization, and image processing algorithm development. Our application is implemented on a laptop (HP630) Intel CORE I3 CUP M370 with 2 Giga byte of RAM and 320 Giga byte hard drive disk HDD and has a 2.40 GHz speed. The minimum required material characteristics for the application are 512 Mega byte of RAM and 80 Giga byte hard drive. To perform tests with our application, we use four databases which are as follows.

- CASIA-Iris V1 [24], CASIA V1, contains 756 images from 108 eyes. For each eye, 7 images are captured in two sessions with a homemade iris camera, where three samples are collected in the first session and four in the second session. All images are stored as BMP format with resolution 320 ∗ 280.
- CASIA-Iris V2 [25] contains 2400 images from 120 eyes. For each eye, 10 images are captured using two different instruments (OKI and Pattek). All images are stored in BMP format with resolution 640∗480. CASIA-Iris V2 contains blurry images with different illuminations and wearing glasses is authorized. The database is available for free on demand.
- FVC 2004 [26] contains four sets DB1_A, DB2_A, DB3_A, and DB4_A. Each of these databases contains 800 fingerprints equivalent of one hundred (100) individuals each having eight (08) impressions. FVC 2004 database is characterized by different fingerprint image qualities. The database is purchased upon request.
- Our proposed database of combined irises and fingerprints made from an equivalent number of irises from CASIA-Iris V2 database and fingerprints from FVC2004 database (50 subjects  ∗  10 images).

For a given database if *c* represents number of classes and *n* represents total number of images per class, then intraclass combinations are calculated as (*n* − 1 × (*n*/2) × *c*) and interclass combinations are calculated as (*c* × (*c* − 1) × *n* × *n*) [27]. For example, for CASIA V1 database, intra-class combinations are worked out as ((7 − 1) × (7/2) × 108) and inter-class combinations are worked out as (108 × 107 × 7 × 7).

For the database CASIA-V2, the intra-class combinations are worked out as ((20 − 1) × (20/2) × 120) = 22800 and interclass are worked out as class combinations (120 × 119 × 20 × 20) = 5,712,000.

For database FVC 2004, intra-class combinations are worked out as ((800 − 1) × (800/2) × 4) = 1,278,400 and inter-class combinations are worked out as (4 × 3 × 800 × 800) = 7680000.

## 6. Experimental Results

The application is divided mainly into three modules.

### 6.1. Iris Recognition Module

**Figure 7:**
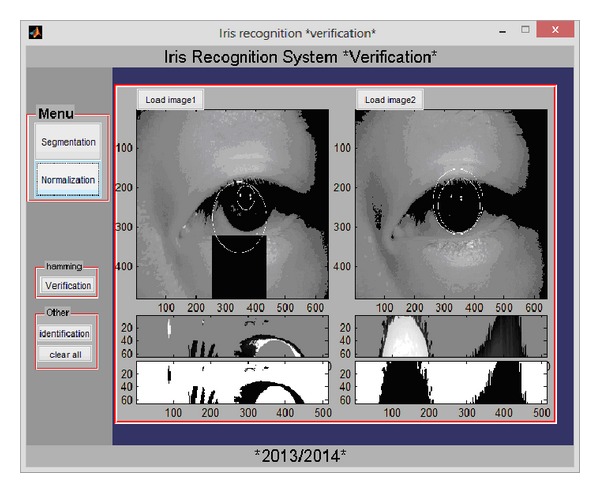
GUI of the verification process in the iris monomodal recognition system.

**Figure 8:**
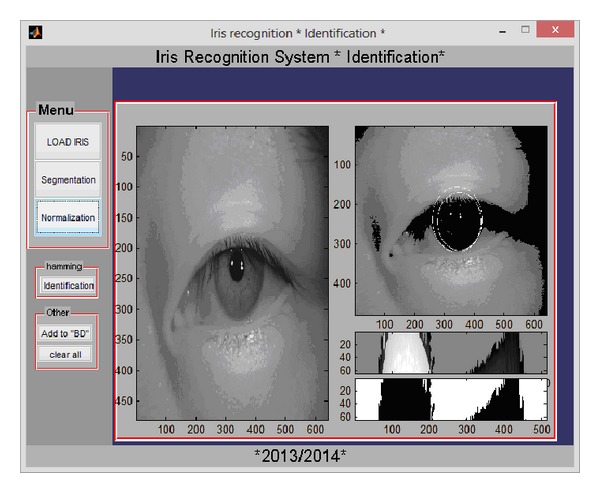
GUI of the identification process in the iris monomodal recognition system.

Both verification and identification processes are implemented. Figures below present the graphical user interfaces GUIs allowing the user to load an iris image from a database and to do segmentation, feature extraction, and either verification (see Figure 7) (the user has to upload another iris image) or identification (see Figure 8) (the system searches similar code in database).

### 6.2. Fingerprint Recognition Module

**Figure 9:**
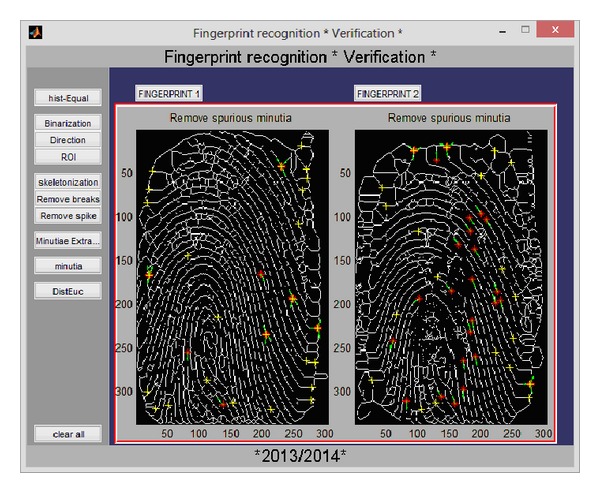
GUI of the verification process in the fingerprint monomodal recognition system.

Like the iris recognition module, both verification and identification processes are implemented.  Figure 9 shows the GUI allowing the user to load two fingerprint images and then visualize the results of each step of the fingerprint recognition algorithm (binarisation, region of interest and the orientation field localisation, the process of image thinning also called skeletonization, the extraction of minutia, the elimination of false minutia, and finally the matching by the Euclidian distance).

**Figure 10:**
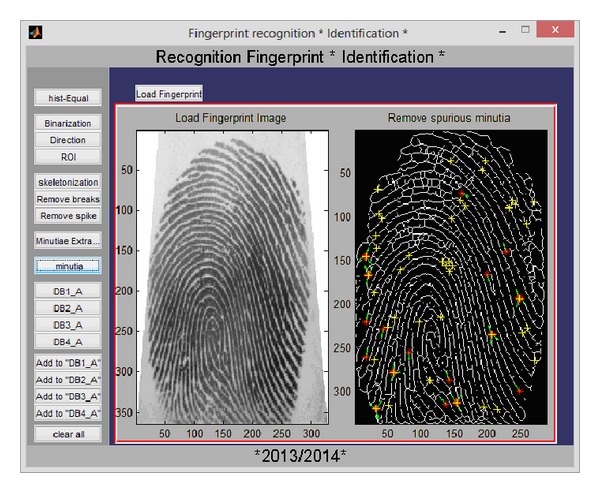
GUI of the identification process in the fingerprint monomodal recognition system.

The identification process in the fingerprint monomodal recognition system consists of matching the generated code from the input image with all codes stored in databases; if the identification failed, the user is asked either to add or not the nonidentified image to a chosen database (see Figure 10).

### 6.3. Combined Iris and Fingerprint Recognition Module

Three matching algorithms are implemented; first is the matching using the fusion of iris and fingerprint by the sum rule, second is the matching of both modalities by the weighted sum rule, and the final is the matching using the fusion by the fuzzy logic if-then rules and the fuzzy inference system.

**Figure 11:**
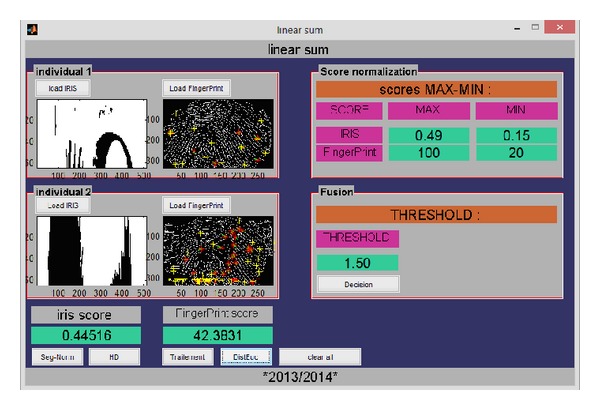
GUI showing the matching using the fusion by the sum rule.

Figure 11 shows the GUI allowing the user to see the verification result of iris and fingerprint combined biometric traits.

**Figure 12:**
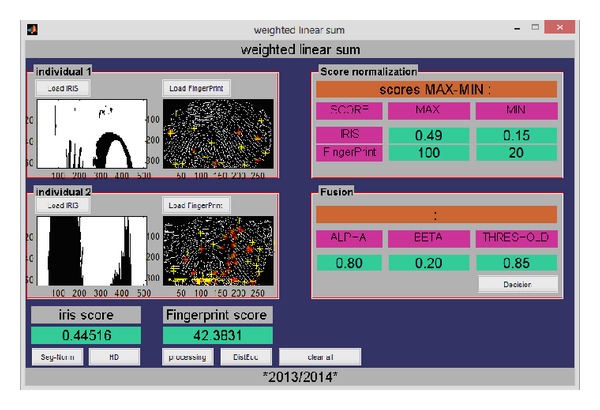
GUI showing the matching using the fusion by the weighted sum rule.

Figure 12 presents the graphical user interface of the recognition module based on the fusion by the weighted sum rule and the score normalization is done prior to fusion using the min-max rule and then the fusion is done; in our experimentation we set *α* to 0.8 for the iris modality and 1 − *α* = 0.2 for the fingerprint modality.

**Figure 13:**
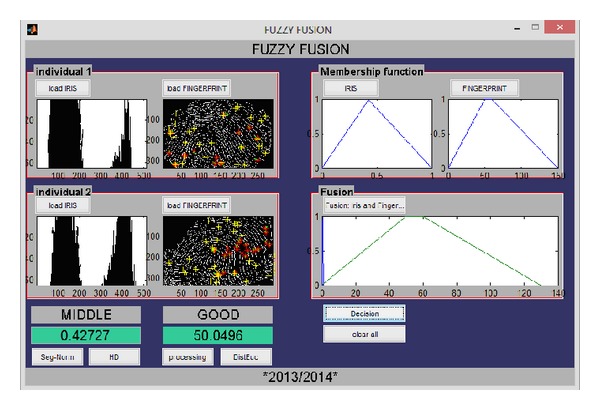
GUI showing the matching using the fusion by the fuzzy inference system.

Figure 13 presents the graphical user interface allowing the user to verify the similarity between two individuals by opening the fingerprint and iris images belonging to each individual, doing feature extraction, and matching operations between the two irises and the two fingerprints, output the matching distances and the decisions of both modalities and then plot the fuzzy membership function for each decision and finally calculate the decision of the combined modalities and plot its fuzzy membership function.

## 7. Performance Evaluation and Comparison

In order to test our proposed schemes for monomodal and multimodal biometric recognition systems and proceed with their evaluation and comparison, we do the following experiments.

**Experiment 1**

Both verification and identification processes are implemented within a monomodal iris recognition system. We use the public code of Masek and Koveski [28] for the verification and we extend it to perform the identification. The feature extractor employed for Iris modality is based on Daugman's approach [29] and was implemented by Masek and Koveski [28]. An Iris code comprising bit streams called Iriscode by Daugman is generated. The hamming distance based matcher provides the matching score. The experiment uses CASIA-V1 iris database.

**Experiment 2**

Both verification and identification processes are implemented within a monomodal iris recognition system. Like Experiment 1, we use the public code of Masek and Koveski [28] for the verification and we extend it to perform the identification. The experiment uses CASIA-V2 iris database.

**Experiment 3**

Both verification and identification processes are implemented within a monomodal fingerprint recognition system and we propose a minutia based fingerprint recognition system using the algorithm of Jagadeesan et al. [16] to localize the region of interest and the orientation field, and the algorithm of Jain et al. [30] for the extraction of minutiae and posttreatment. Matching is based on Euclidian distance. The experiment uses FVC2004 fingerprint database.

**Experiment 4**

Only verification process is implemented within a multimodal biometric recognition system of combined iris and fingerprint using the sum rule based matching. The experiment uses an equivalent number of images from CASIA Iris-V2 and FVC2004 fingerprint databases (5 from each modality  ∗  50 subjects).

**Experiment 5**

Only verification process is implemented within a multimodal biometric recognition system of combined iris and fingerprint using the weighted sum rule based matching. The experiment uses an equivalent number of images from CASIA Iris-V2 and FVC2004 fingerprint databases (5 from each modality  ∗  50 subjects).

**Experiment 6**

Only verification process is implemented within a multimodal biometric recognition system of combined iris and fingerprint using our proposed fuzzy logic based matching. The experiment uses an equivalent number of images from CASIA Iris-V2 and FVC2004 fingerprint databases (5 from each modality  ∗  50 subjects). For all the tests, we use the FVC2004 testing protocol [26] for the fingerprint and iris recognition modules.

The fingerprint testing protocol is described as follows.

- Genuine recognition attempts: the template of each impression is matched against the remaining impressions of the same individual, but avoiding symmetric matches.
- Impostor recognition attempts: the template of the first impression is matched against the first impression of the remaining individuals, but avoiding symmetric matches.

The iris testing protocol is described as follows.

- First the database is divided into two parts: 40% of the database is reserved to enrolment in order to estimate the classifier parameters, and 60% of the database is used to test and validate the classifier.
- Genuine recognition attempts: the template of each iris is matched against the remaining irises of the same individual, but avoiding symmetric matches.
- Impostor recognition attempts: the template of the first iris is matched against the first iris of the remaining individuals, but avoiding symmetric matches.

For experiments using fusion module, tests are conducted on a set of images belonging to 50 subjects having five fingerprint images from FVC 2004 fingerprint database and five iris images from CASIA-Iris V2 database.

### 7.1. Time Execution Comparison

The values presented in Table 1 are results of execution time using CASIA V1, CASIA V2, and FVC 2004 databases and MATLAB 7.10.0(R2010a) programming tool.

We note that the fastest system in terms of matching recognition time is the monomodal fingerprint recognition system and this is mainly due to the use of the Euclidean distance in the matching phase (see Table 1).

Our experimental results shows that the fuzzy logic method for the matching scores combinations at the decision level is the best in terms of matching time followed by the classical weighted sum rule and the classical sum rule in order.

### 7.2. Results in Terms of Error Rates FRR, FAR, and EER

The false reject rate (FRR), also known as type I error, measures the probability of an enrolled individual not being identified by the system. The false accept rate (FAR), also known as type II error, measures the probability of an individual being wrongly identified as another individual [28]. According to the statistical analysis in which we have calculated the inter-class and the intra-class thresholds using the above experiments, whose values minimize the rates of false acceptance and false rejection, we have estimated the best thresholds for minimal error rates for each experiment. See Tables 2, 3, 4, 5, and 6 (values in bold are best FAR and FRR for corresponding threshold).

Unlike Experiments 1–5, we have no thresholds in Experiment 6 using our proposed fuzzy matching fusion algorithm, but we have decisions.  Table 7 shows an example of its intra-class and inter-class distributions.

According to our proposed fusion by fuzzy matching scheme based on if-then rules explained earlier, the results are either excellent or very good, or good or medium, or bad or very bad. The decision “medium” means that

- either the fingerprint recognition result is “bad” and the iris recognition result is “medium",
- or the fingerprint recognition result is “good” and the iris recognition result is “bad,” If we accept the decision “medium” as being genuine recognition of the individual so we achieve FAR = 0.16 and FRR = 0.0. If we reject the decisions “medium” as being imposter attempts so we achieve FAR = 0.0 and FRR = 0.05.

Experimental results show that the equal error rate calculated by Experiment 6 (our proposed fuzzy matching fusion) is EER = 0.038.

**Figure 14:**
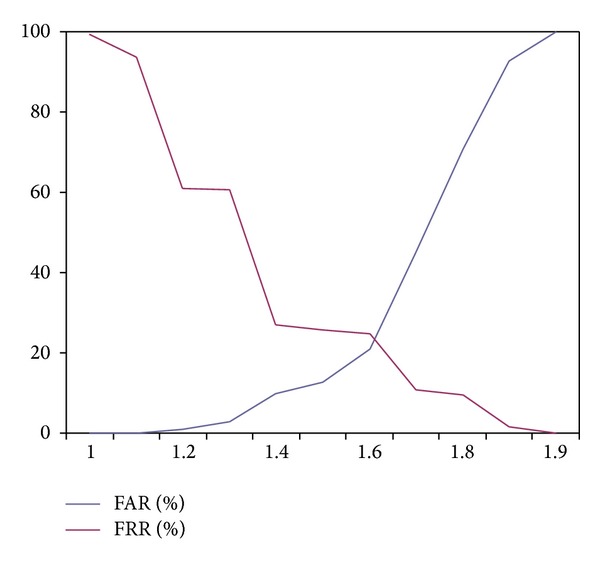
FAR and FRR using Experiment 4 (iris and fingerprint fusion based sum rule matching).

Figure 14 represents the plot of FAR and FRR using Experiment 4 (iris and fingerprint fusion based sum rule matching).

**Figure 15:**
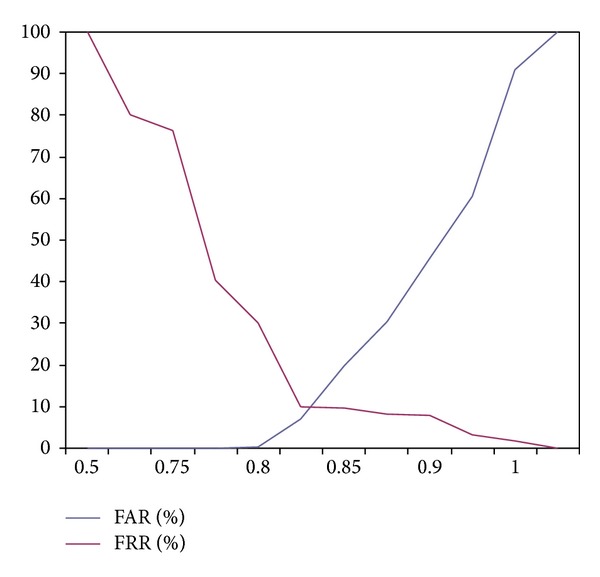
FAR and FRR using Experiment 5 (iris and fingerprint fusion based weighted sum rule matching).

As mentioned by Figure 14, the equal error rate for Experiment 4 is 1.55.  Figure 15 represents the plot of FAR and FRR using Experiment 5 (iris and fingerprint fusion based weighted sum rule matching).

Experimental results show that Experiment 5 achieves an equal error rate of 0.83. Here we compare the equal error rates of all the experiments we have carried out (see Table 8).

Table 8 presents an equal error rate comparison of the different recognition methods we have implemented; we see that Experiment 6 performing iris and fingerprint fusion by our proposed fuzzy logic matching method is the best followed by the weighted sum rule fusion based matching and finally the sum rule fusion based matching.

### 7.3. Results in Terms of Accuracy

Table 9 presents accuracy comparison of all the experiments we have conducted.

In biometry, the system accuracy is calculated as follows:

(7) $$\begin{array}{c} \mathrm{AC}=100-\frac{\text{FRR}+\text{FAR}}{2}. \end{array}$$

According to the results presented in Table 9, we conclude that the accuracy of the method of the fusion of decisions by fuzzy logic is better than that of the other techniques. This comparison is done to illustrate the fact that the proposed system provides improved results as compared to the results from the individual unimodal systems and the results from the implemented multimodal systems using traditional matching.

### 7.4. Comparison with Related Works in the Current Literature

Table 10 presents a comparison of the different recognition methods proposed and implemented in the current literature; we see that Experiment 6 performing iris and fingerprint fusion by our proposed fuzzy logic matching method is the better in terms of error rates than the other presented systems, and the matching time is comparable to that of Gawande et al.'s [20] system.

## 8. Conclusion

The objective of this research is the introduction of a novel matching approach for multimodal biometric recognition based on fuzzy logic. The biometric traits used in our work are iris and fingerprint.

In this paper a novel combination of iris and fingerprint biometrics is presented in order to achieve best compromise between a zero FAR and its corresponding FRR; in our approach, iris trait has more weight in fusion with fingerprint and the system decision is made to have more intermediate values between bad and good recognition and the weight is simply an appreciation we assign to the matching distance for each single biometric set by fuzzy membership function; the fuzzy inference system mimics our human thinking and this is mainly the reason we get enhanced results. The contribution of this research is threefold, first designing and implementing monomodal systems for the biometric recognition of iris and fingerprint, second designing and implementing a multimodal biometric system of combined iris and fingerprint using the previous monomodal systems with three different matching algorithms, two classical matching algorithms and our proposed one based on fuzzy logic, and third carrying out exhaustive and intensive tests on the iris and fingerprint databases using the proposed recognition schemes to conclude at the end the best one. At last, a comparison of the achieved results with similar works in the current literature is given and our experimental results are the best in terms of matching time, error rates, and accuracy.

The normalization of scores is required prior to the fusion only for the classical fusion. Fusion by fuzzy logic does not require normalization of scores; only decisions are used by the fuzzy inference system. Three matching algorithms are used: the classical sum rule matching, the weighted sum rule matching, and our proposed matching with fuzzy logic. These fusion methods act on two different levels, namely,

- the score fusion level: in which we implemented the method of the classical linear sum rule of iris and fingerprint scores and method of the weighted linear sum which give weight to iris and fingerprint sores,
- the decision fusion level: where we have designed and implemented a fuzzy matching technique after converting iris and fingerprint scores to fuzzy sets (this conversion is called fuzzification), the fuzzy inference system produced fuzzy results (bad recognition, or very bad, or medium, or good, or very good or excellent).

Our proposed fuzzy matching algorithm assigns a specific appreciation to each decision according to the best threshold minimizing both FRR and FAR. The fuzzy if-then rules produce decisions according to the matching distance calculated for each modality. For experiments using fusion module, tests are conducted on a set of images belonging to 50 subjects having five fingerprint images from FVC 2004 fingerprint database and five iris images from CASIA-Iris V2 database. Experimental results achieved best compromise between FRR and FAR (0% FAR and 0.05% FRR) with accuracy 99.975% and EER equal to 0.038 and matching time equal to 0.1754s.

This work allowed us to draw the following conclusions.

- The multimodal fusion gives better results than using a single matching recognition module for iris or fingerprint.
- Matching with fuzzy logic provides enhanced recognition results followed by the classical weighted sum rule and the classical sum rule in order.

This work belongs to biometric security domain. It gives solution to the problem of person identification with lower errors, high accuracy, and less complexity of the system. The fuzzy logic inference system used in the matching phase is simple and robust at the same time. Inperspective, we suggest that more attention should be given to the quality enhancement of the input biometric data in order to decrease some biometric system failures like failure to enroll and failure to match.

## Figures and Tables

**Table 1:** Matching time comparison.

	Verification	Identification
Iris recognition using CASIA Iris-V1	0.138	0.1797
Iris recognition using CASIA-Iris-V2	0.155	0.298
Fingerprint recognition using FVC 2004	0.087	0.15876
Fusion by sum rule	0.256	/
Fusion by weighted sum rule	0.2487	/

Proposed fusion by fuzzy logic	0.1754	/

**Table 2:** Best FAR, FRR, and corresponding threshold for Experiment 1.

Threshold	FAR (%)	FRR (%)
0.20	0.000	99.047
0.25	0.000	82.787
0.30	0.000	37.880
0.35	0.000	5.181
**0.40**	**0.005**	**0.238**
0.45	7.599	0.000
0.50	99.499	0.000

**Table 3:** Best FAR, FRR, and corresponding threshold for Experiment 2.

Threshold	FAR (%)	FRR (%)
0.20	0.000	99.90
0.25	0.000	95.80
0.30	0.000	57.78
0.35	0.000	20.43
0.40	0.01	9.89
**0.45**	**0.099**	**4.09**
0.50	99.499	0.000

**Table 4:** Best FAR, FRR, and corresponding threshold using Experiment 3.

Threshold	FAR (%)	FRR (%)
10	0.000	99
20	0.005	95.80
30	0.10	70.29
40	0.60	56.78
50	10	30.89
60	13.43	28.78
70	13.95	26.77
**80**	**14.01**	**15.98**
90	67.87	50.76
100	90.89	12.67

**Table 5:** Best FAR, FRR, and corresponding threshold for Experiment 4.

Threshold	FAR (%)	FRR (%)
1.0	0.000	99.30
1.10	0.08	93.56
1.20	0.90	60.89
1.30	2.7	60.58
1.40	10	26.89
**1.50**	**12.83**	**25.78**
1.60	20.95	24.77
1.70	45.01	10.98
1.80	70.87	9.60
1.85	92.81	1.67
1.90	99.98	000

**Table 6:** Best FAR, FRR, and corresponding threshold for Experiment 5.

Threshold	FAR (%)	FRR (%)
0.7	0.000	80.0
0.75	0.05	76.25
0.78	0.07	40.23
0.80	0.2	30
**0.83**	**7**	**10**
0.85	20	9.78
0.88	30.47	8.25
0.90	45.54	8
0.95	60.541	3.25
1.00	90.8	1.87
1.5	99.99	000

**Table 7:** Example of intraclass and interclass distributions using Experiment 6 (our proposed fuzzy matching fusion).

	Individual 1			Individual 3		
Individual 1	Good	Good	Medium	Bad	Bad	Bad
	Very good	Good	Medium	Very bad	Very bad	Bad

Individual 2	Very bad	Bad	Very bad	Very bad	Medium	Bad
	Bad	Bad	Bad	Bad	Bad	Bad

Individual 3	Bad	Bad	Bad	Good	Good	Good
	Bad	Very bad	Very bad	Medium	Good	Good

Individual 4	Bad	Bad	Bad	Bad	Bad	Bad
	Bad	Bad	Very bad	Very bad	Very bad	Bad

Individual 5	Bad	Medium	Bad	Very bad	Bad	Bad
	Bad	Bad	Very bad	Bad	Bad	Bad

**Table 8:** Equal error rate comparison.

Experiment	EER
Experiment 1: iris (CASIA-Iris V1)	0.40
Experiment 2: iris (CASIA-Iris V2)	0.45
Experiment 3: fingerprint (FVC 2004)	0.5
Experiment 4: iris + fingerprint (sum rule fusion based matching)	1.55
Experiment 5: iris + fingerprint (weighted sum rule fusion based matching)	0.83
Experiment 6: iris + fingerprint (fuzzy logic fusion based matching)	0.038

**Table 9:** Accuracy comparison of all the implemented systems.

Experiment	Accuracy %
Experiment 1: iris (CASIA-Iris V1)	99.87
Experiment 2: iris (CASIA-Iris V2)	97.9
Experiment 3: fingerprint (FVC 2004)	85
Experiment 4: iris + fingerprint (sum rule fusion based matching)	80.69
Experiment 5: iris + fingerprint (weighted sum rule fusion based matching)	91.5
Experiment 6: iris + fingerprint (fuzzy logic fusion based matching)	99.975

**Table 10:** Performance comparison with related systems.

Author and reference	Level of fusion	Database	Extractor	Matcher	Results
Kankrale and Sapkal 2012 [9]	Feature extraction	500 images of 50 subjects (from CASIA-fingerprint V5 and CASIA-iris V1)	Minutia based extractor + Daugman's iris extractor	AND rule	FAR = 0%, FRR = 5.12% Match time = 3.56 s

Gawande et al. 2012 [20]	Feature extraction	500 images of 50 subjects	1D log Gabor filter for both modalities	HD (hamming distance)	FAR = 0%, FRR = 4.3% Match time = 0.14 s

Abdolahi et al. 2013 [7]	Decision	Not given	Modified minutia based extractor + iris extractor not given	Fuzzy rules + weighted code	FAR = FRR = 2% Match time not given.

Our proposed fuzzy logic based matching scheme	Decision	500 images of 50 subject (from FVC 2004 and CASIA-Iris V2)	Minutia based extractor + Daugman's iris extractor	Fuzzy if-then rules	FAR = 0% FRR = 0.05% Match time = 0.1754 s
